# Effectiveness and safety of anti-PD-1 monotherapy or combination therapy in Chinese advanced gastric cancer: A real-world study

**DOI:** 10.3389/fonc.2022.976078

**Published:** 2023-01-05

**Authors:** Tao Li, Tingting Liu, Lei Zhao, Lu Liu, Xuan Zheng, Jinliang Wang, Fan Zhang, Yi Hu

**Affiliations:** ^1^ Graduate School, Medical School of Chinese People's Liberation Army (PLA), Beijing, China; ^2^ Department of Oncology, The First Medical Center, Chinese People's Liberation Army (PLA) General Hospital, Beijing, China; ^3^ Chinese People's Liberation Army (PLA) Key Laboratory of Oncology, Key Laboratory for Tumor Targeting Therapy and Antibody Drugs, Ministry of Education, Beijing, China; ^4^ Department of Pulmonary and Critical Care Medicine, the Second Medical Center, Chinese People's Liberation Army (PLA) General Hospital, Beijing, China; ^5^ Institute of Translational Medicine, Chinese People's Liberation Army (PLA) General Hospital, Beijing, China; ^6^ Department of Nutrition, The First Medical Center, Chinese People's Liberation Army (PLA) General Hospital, Beijing, China; ^7^ Department of Oncology, The Fifth Medical Center, Chinese People's Liberation Army (PLA) General Hospital, Beijing, China

**Keywords:** anti-PD-1, gastric cancer, real-word study, Chinese, efficacy and safety analyses

## Abstract

**Purpose:**

Gastric cancer (GC) is one of the most frequently diagnosed cancers and one of the leading causes of cancer deaths worldwide, especially in eastern Asia and China. Anti-PD-1 immune checkpoint inhibitors, Pembrolizumab and Nivolumab, have been approved for the treatment of locally advanced or metastatic gastric or gastroesophageal junction cancer (GC/GEJC). Our study evaluated the effectiveness and safety of anti-PD-1-based treatment (monotherapy or combination therapy) in Chinese patients with advanced or metastatic GC/GEJCs in a real-world setting.

**Methods:**

A retrospective cohort study was conducted, and 54 patients from May 31, 2015, to May 31, 2021, were included in our analysis, including 19 patients treated with anti-PD-1 monotherapy and 35 patients treated with anti-PD-1 combination therapy. Demographic and clinical information were evaluated. Clinical response, survival outcomes, and safety profile were measured and analyzed.

**Results:**

Overall, the median overall survival (mOS) was 11.10 months (95% CI, 7.05–15.15), and the median progression-free survival (mPFS) was 3.93 months (95% CI, 2.47–5.39). Of the patients, 16.7% achieved a clinical response, and 72.2% achieved disease control. Prolonged overall survival (OS) and progression-free survival (PFS) and increased clinical response were observed in the combination group compared with the monotherapy group, although statistical significance was not reached. In subgroups with live metastases or elevated baseline neutrophil-to-lymphocyte ratio (NLR) levels, combination therapy outperformed anti-PD-1 alone in survival outcomes. Patients treated with anti-PD-1 monotherapy (n = 5, 26.3%) had fewer treatment-related adverse events (TRAEs) than those in the combination group (n = 22, 62.9%). There were also fewer patients with TRAEs of grades 3–5 with monotherapy (n = 2, 10.5%) than with combination therapy (n = 7, 20.0%). Pneumonitis in three patients was the only potential immune-related adverse event reported.

**Conclusions:**

Anti-PD-1-based monotherapy and combination therapy showed favorable survival outcomes and manageable safety profiles in advanced or metastatic GC/GEJCs. In clinical treatment, immunotherapy should be an indispensable choice in the treatment strategy for GC/GEJC. Patients with a heavy tumor burden and more metastatic sites might benefit more from combination therapy. Elderly patients and patients with more treatment lines or high Eastern Cooperative Oncology Group (ECOG) performance scores might be more suitable for immune monotherapy, and some clinical benefits have been observed.

## 1 Introduction

Gastric cancer (GC) is one of the most frequently diagnosed cancers and one of the leading causes of cancer deaths worldwide (1). It is more prevalent in eastern Asia and China. The estimated incidence and mortality of GC in 2015 were 679,100 and 498,000, respectively, ranking as the second most common cancer (2, 3). Most patients are diagnosed at an advanced stage, with a 5-year survival rate of only 33% (4). Currently, the first-line treatment for advanced GC patients is primarily platinum plus fluoropyrimidine, or trastuzumab in combination with it, for HER2-overexpressing tumors (5, 6). Preferred treatment modalities for second-line or beyond include ramucirumab plus paclitaxel or monotherapy of docetaxel, paclitaxel, irinotecan, or ramucirumab (5).

In recent years, immunotherapy has been a revolutionary treatment strategy for advanced cancers. Immune checkpoint blockades (ICBs), including antibodies to Programmed Death Receptor 1 (PD-1) or its ligand (PD-L1), are now standard therapies for a range of solid tumors as approved by the Food and Drug Administration (FDA) (7). Several clinical trials have shown that some GC patients could benefit from anti-PD-1/PD-L1 antibody therapy, indicating that ICBs are a potential treatment option for GC. For ICBs’ monotherapy as third-line, pembrolizumab (a humanized anti-PD-1 IgG4 monoclonal antibody) was approved by the FDA for the treatment of locally advanced or metastatic gastric or gastroesophageal junction cancer (GC/GEJC) patients with PD-L1 positive tumors (Combined Positive Score (CPS) ≥1). This is based on the data of KEYNOTE-059, a single-arm study (8). In ATTRACTION-2, a phase III clinical trial comparing nivolumab with a placebo in Asian patients who were heavily pretreated, nivolumab (a humanized anti-PD-1 IgG4 monoclonal antibody) led to improved overall survival (OS) and progression-free survival (PFS) and was, in general, well tolerated (9). However, in another phase III clinical trial conducted on a global scale (JAVELIN gastric 300), avelumab (a humanized anti-PD-L1 IgG1 monoclonal antibody) did not improve OS or PFS compared with chemotherapy in the total population (including 25.4% Asian patients) as a third-line treatment (10). For first- or second-line treatment, only three single-arm studies showed a 7%–22% objective response rate (ORR) from ICB monotherapy (11–13), and two phase III trials reported no significant clinical benefit from pembrolizumab monotherapy compared with chemotherapy (14, 15). Because of the modest benefit of ICB monotherapy, co-administration with another therapeutic agent provides a potential solution to enhance treatment effectiveness. However, in KEYNOTE-062, the only phase III study investigating pembrolizumab plus chemotherapy as first-line treatment of advanced GC/GEJCs, the arm given pembrolizumab plus chemotherapy did not exhibit significantly prolonged OS than those taking chemotherapy alone (15). Several phase I-II studies preliminarily showed that ICBs combined with chemotherapy, targeted, or antiangiogenic agents presented improved ORR (16–18). Most of the studies above were conducted on Caucasian patients, while little was known about how Asian GC/GEJCs patients, either treatment-naïve or previously treated, would respond to ICBs therapy. This represents an unmet medical need since Asians are known to be heavily burdened with GC/GEJCs. Moreover, the populations in the clinical trials described above are usually highly selected. It remains inconclusive whether patients with an Eastern Cooperative Oncology Group (ECOG) performance score 1, patients with brain metastases, or patients over 70 years old could benefit from ICBs’ treatment since they are usually excluded from interventional clinical trials. To explore whether ICBs could bring clinical benefit to Asian GC patients, especially in China, we conducted a real-world study to examine the safety and anti-tumor effectiveness of anti-PD-1 monotherapy and combination therapy in advanced GC patients. To our knowledge, it was one of the first real-world clinical studies of immunotherapy for GC/GEJC in China. Through this study, it was hoped that real-world studies could play a complementary role in clinical trials and provide more evidence-based medical support for immunotherapy for GC/GEJC in Asia, especially in China.

## 2 Materials and methods

### 2.1 Study design and participants

This study conducted a retrospective analysis to evaluate the effectiveness and safety profile of anti-PD-1 treatment in a real-world setting. Consecutive GC/GEJCs patients treated at the Department of Oncology, Chinese People’s Liberation Army (PLA) General Hospital from May 31, 2015, to May 31, 2021, were reviewed and screened. Inclusion criteria were: 1) pathologically confirmed GC/GEJCs; 2) advanced or metastatic disease or recurrence after curative surgery; and 3) administration of nivolumab or pembrolizumab as monotherapy or combination therapy. The ethics committee of PLA General Hospital approved this study according to the ethical standards of the Declaration of Helsinki and its subsequent amendments (Ethical approval number: S2020-284-01).

Clinicopathological characteristics were reviewed and collected. Two physicians independently extracted and recorded demographic and clinical information, followed by confirmation by a third physician in case of inconsistency. Data were collected from the following sources: 1) Chinese PLA General Hospital inpatient and outpatient records, including doctors’ notes, radiographic reports, and biopsy results; 2) patient or family interviews. Complete blood cell counts of eligible patients, covering neutrophils, lymphocytes, thrombocytes, and erythrocytes, were also collected. NLR was defined as the absolute neutrophil count divided by the entire lymphocyte count in peripheral blood, collected before the initiation of anti-PD-1 treatment. The platelet-to-lymphocyte ratio (PLR) was defined as the absolute thrombocyte count divided by the absolute lymphocyte count. Median NLR and PLR were adopted as cutoffs in our analysis.

### 2.2 Study objectives

The primary endpoint was OS, defined as the time from the first dose of anti-PD-1 to death from any cause. Secondary endpoints included PFS (defined as the time from treatment initiation to the first documented disease progression or death), ORR (defined as the proportion of patients with confirmed complete response or partial response), and disease control rate (DCR), which was defined as the proportion of patients with confirmed complete response, partial response, or stable disease, duration of response, and the maximum percentage change from baseline for the sum of diameters of target lesions. All endpoints were evaluated according to RECIST (version 1.1) guidelines (19). Follow-up imaging reports were reviewed by two radiologists independently. The director of the imaging center further verified any discrepancies. Data for patients without disease progression or death events were censored at the time of the last follow-up. Adverse events (AE) were evaluated and graded according to the National Cancer Institute Common Terminology Criteria for Adverse Events (version 5.0) (20). Treatment-related adverse events (TRAEs) and immune-related AEs were graded and recorded. All patients were followed up until December 2021, or death.

### 2.3 Ethics approval and consent to participate

All participants in this study signed informed consent forms. The ethics committee of the PLA General Hospital approved this study according to the ethical standards of the Declaration of Helsinki and its subsequent amendments (Ethical approval number: S2020-284-01).

### 2.4 Statistical analysis

Categorical characteristics and objective response were compared between treatment groups with the chi-square test or Fisher’s exact test. Continuous variables were compared using the Mann-Whitney U test. Kaplan-Meier survival analysis was used to assess OS and PFS, and the log-rank test was used to compare groups. The Hazard Ratio (HR) was estimated using the Cox proportional hazard model. In the multivariable Cox regression model, variables with a *P*-value <0.10 in the univariable Cox regression or acting as clinically relevant factors were adjusted. All *P*-values are two-tailed, and a *P*-value <0.05 was considered statistically significant. The SPSS statistical package (SPSS 20, SPSS, Chicago, IL, USA) was used for all statistical analyses.

## 3 Results

### 3.1 Patients’ characteristics and treatment

Fifty-four patients received anti-PD-1-based therapy during their course of disease and were identified and included in our analysis, including 19 patients treated with anti-PD-1 monotherapy and 35 patients treated with anti-PD-1 combined with chemotherapy (XELOX, SOX, and mFOLFOX6) targeted therapy or anti-CTLA-4 (a monoclonal antibody targeting cytotoxic T-lymphocyte-associated antigen-4). Patients’ clinicopathological characteristics are summarized in **Table 1**. The median age of the recruited patients was 58 years in the monotherapy group and 59 years in the combination therapy group, where 2 (10.5%) and 9 (25.7%) patients received immunotherapy at the age of over 65 in the two groups, respectively. In the two groups, 11 (57.9%) and seven patients (20.0%) had an ECOG performance score of 2-4, respectively. Fewer patients in the monotherapy group received anti-PD-1 as first-line treatment than in the combination group (5.3% vs. 37.1%, *P* = 0.01), while more patients in the monotherapy groups were treated with anti-PD-1 as 4^th^ line or later therapy (36.8% vs. 11.4%, *P* = 0.04), respectively. Due to the long period, people initially did not realize that PD-L1 status was vital for immunotherapy when the drug was marketed, so only a small number of patients completed the test. Only 5/19 and 8/35 patients from monotherapy and combination therapy completed the PD-L1 test respectively (**Table 1**).

**Table 1:** Baseline demographic and clinical characteristics of GC/GEJC patients.

	Univariate Cox model			Multivariate Cox model		
Characteristics	HR	95%CI	P value	HR	95%CI	P value
**Gender**	0.61	0.31-1.24	0.17			
Male vs female						
**Age**	1.12	0.49-2.57	0.79			
≥65 vs <65						
**EOCG performance status**	0.28	0.11-0.56	**<0.001**	0.19	0.09-0.40	**<0.001**
0-1 vs 2-4						
**Primary tumor site**	0.38	0.05-2.78	0.34			
Gastric vs Gastro-oesophageal junction						
**TNM stage**	0.35	0.08-1.45	0.15			
III vs IV						
**Metastasis**	2.34	0.55-9.89	0.25			
Yes vs No						
**Organs with metastases**	1.32	0.68-2.53	0.41			
≤2 vs >2						
**Ascites**	3.27	1.61-6.65	**0.001**	4.36	2.02-9.44	**<0.001**
Yes vs No						
**Lymph node metastases**	1.52	0.54-4.29	0.43			
Yes vs No						
**Peritoneum metastases**	1.29	0.66-2.52	0.46			
Yes vs No						
**Liver**	0.60	0.31-1.16	0.13			
Yes vs No						
**Treatment regimen**	0.69	0.36-1.33	0.26	1.02	0.46-2.28	0.97
Com vs mono						
**Treatment lines**	0.84	0.44-1.62	0.61			
1-2 vs >2						
**Elevated LDH**	1.37	0.57-3.31	0.48			
Yes vs No						
**NLR**	2.19	1.18-4.05	**0.01**	1.65	0.65-4.21	0.29
>median vs <median						
**PLR**	2.49	1.33-4.65	**0.004**	1.96	0.74-5.16	0.17
>median vs <median						
**Adverse event**	0.80	0.41-1.55	0.50			
Yes vs No						

ECOG, Eastern Cooperative Oncology Group.

LDH, lactate dehydrogenase.

NLR, neutrophil to lymphocyte ratio.

PLR, Platelet to lymphocyte ratio.

The meaning of the bold values was P value <0.05 and was considered statistically significant.

### 3.2 Treatment outcome

**Figure 1:**
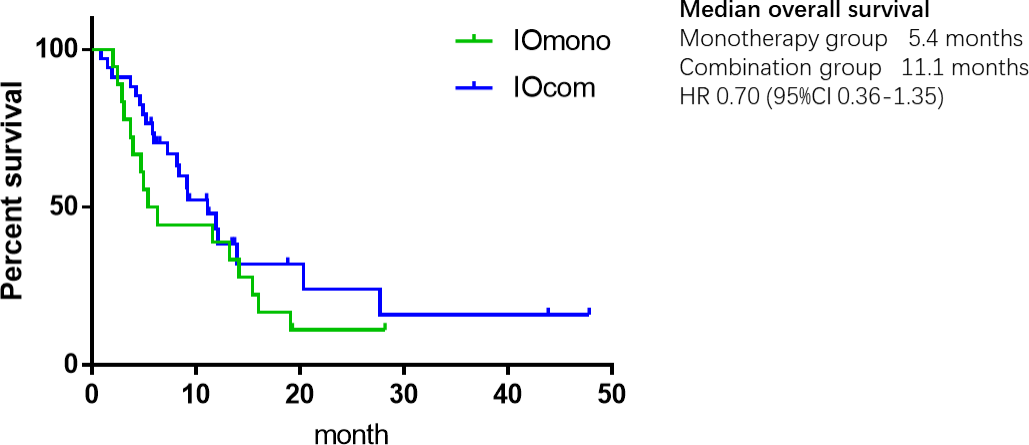
Kaplan-Meier plot of overall survival.

**Figure 2:**
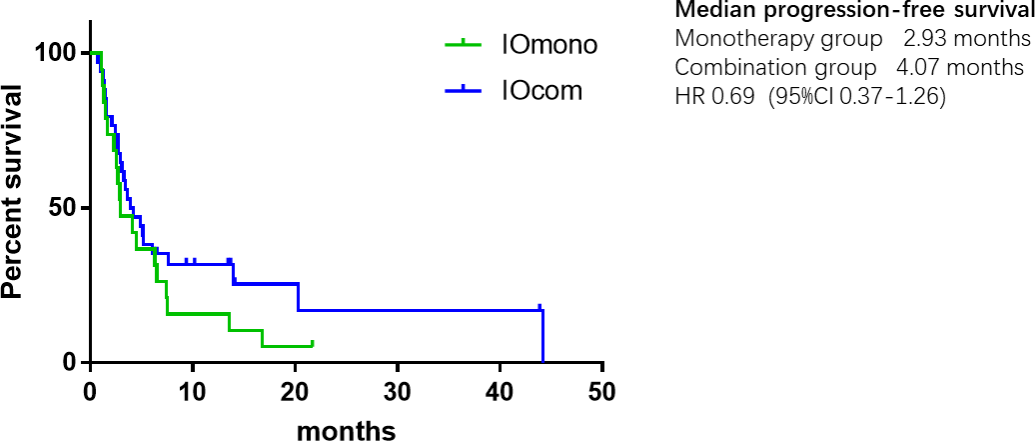
Kaplan-Meier plot of progression-free survival.

As of the data cut-off date of May 31, 2021, the median follow-up was 9.40 months, ranging from 0.80 to 43.80 months. 44 (81.5%) progression events and 37 (68.5%) deaths occurred during the follow-up period. In the overall cohort, the mOS was 11.10 months (95% CI, 7.05–15.15), and the mPFS was 3.93 months (95% CI, 2.47–5.39). Prolonged mOS was observed in the patients receiving combination therapy (11.10 months; 95% CI, 7.18–15.03), and that was over those taking anti-PD-1 alone (5.40 months; 95% CI, 2.64–8.17). However, the difference in OS between the two groups was not statistically significant, possibly due to the small sample size (HR = 0.70, 95% CI, 0.36–1.35, *P* = 0.29) (**Figure 1**). Likewise, the mPFS of the combination group at 4.07 months (95% CI, 1.83–6.03) was non-significantly longer than that of the monotherapy group at 2.93 months (95% CI, 0.85–5.01) (HR = 0.69, 95% CI, 0.37–1.26, *P* = 0.22) (**Figure 2**).

**Figure 3:**
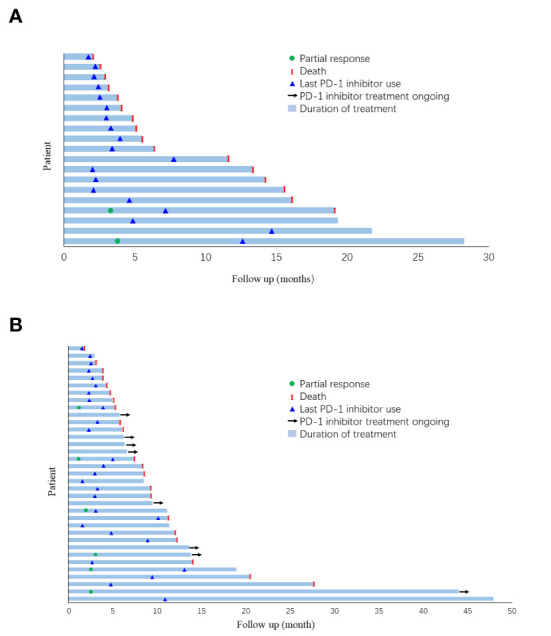
Duration of follow-up and outcome for each patient in **(A)** monotherapy group and; **(B)** combination group.

In the overall population, for patients who received anti-PD-1-based therapy as first- or second-line treatment, the mOS and mPFS were, respectively, 11.10 months (95% CI, 6.36–15.85) and 3.43 months (95% CI, 2.20–4.66). In patients treated with anti-PD-1-based therapy as the third-line or beyond, the mOS and mPFS were 9.13 months (95% CI, 2.22–16.04) and 4.20 months (95% CI, 1.60–6.80), respectively. There was no statistical difference between the monotherapy and combination groups regarding OS or PFS upon stratification by treatment lines (**Supplementary Figures 1**–**4**). The duration of treatment and outcomes for each patient were specified in **Figure 3**.

**Figure 4:**
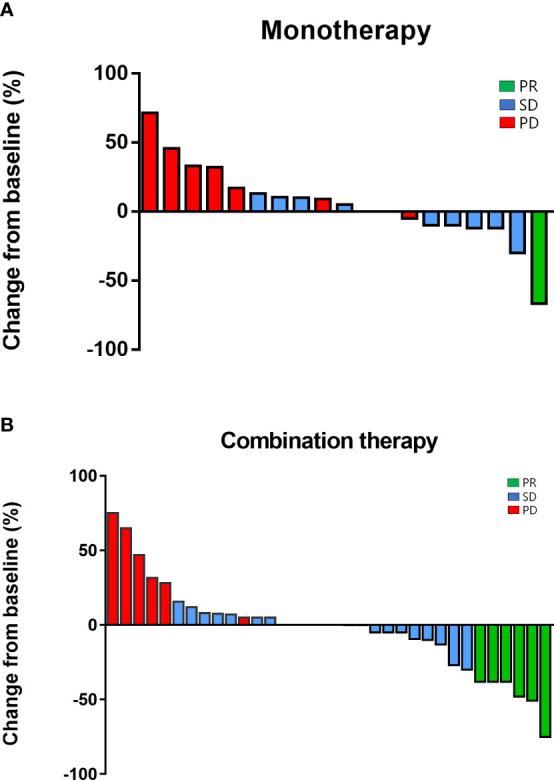
Regression of target lesions from baseline in each patient in **(A)** the monotherapy group and; **(B)** the combination group.

Response rates for the overall population, the monotherapy group, and the combination arm were summarized in **Table 2**. ORR and DCR were 16.7% (95% CI, 6.4–26.9%) and 72.2% (95% CI, 59.9–84.6%) of the overall cohort, respectively. ORR tended to be higher in the combination group (20.0%, 95% CI, 8.4–36.9%) than in the monotherapy group (10.5%, 95% CI, 1.9–29.6%), but the difference was not statistically significant (*P* = 0.47) with the limited sample size. DCR was 63.2% and 77.1% in the monotherapy and combination groups, respectively (*P* = 0.35). Concerning the best overall responses, 36.8% (7/19) of the patients given monotherapy and 40.0% (14/35) of the patients on combination treatment achieved a decrease from baseline in the sum of their target lesions (**Figure 4**).

**Table 2:** Objective tumor response of GC/GEJC patients.

	Monotherapy	Combination therapy	
	(n=19)	(n=35)	P value
**Gender**			1.00
Male	11(57.9%)	21(60.0%)	
Female	8(42.1%)	14(40.4%)	
**Age**			0.29
Median (range), years	58(34–81)	59(24-86)	
<65	17(89.5%)	26(74.3%)	
≥65	2(10.5%)	9(25.7%)	
**ECOG performance status**			**0.01**
0	2(10.5%)	21(60.9%)	
1	6(31.6%)	7(20.0%)	
2	6(31.6%)	5(14.3%)	
3	4(21.1%)	2(5.7%)	
4	1(5.3%)	0	
**TNM stage**			0.61
III	2(10.5%)	2(5.71%)	
IV	17(89.5%)	33(94.3%)	
**Tumor site**			0.54
Gastric	19(100%)	32(91.4%)	
Gastro-oesophageal junction	0	3(8.6%)	
**Organs with metastases**			0.25
≤2	7(36.8%)	20(57.1%)	
>2	12(63.2%)	15(42.9%)	
**Site of metastases**			
Lymph node	18(94.7%)	29(82.9%)	1.00
Peritoneum	13(68.4%)	18(52.9%)	0.26
Liver	5(26.3%)	22(64.7%)	**0.02**
Bone	4(21.1%)	8(23.5%)	1.00
Lung	3(15.8%)	4(11.4%)	0.68
Adrenal	1(5.3%)	3(8.8%)	1.00
**Previous anticancer therapies for locally advanced/metastatic disease**			
0	1(5.3%)	13(37.1%)	**0.01**
1	7(36.8%)	14(40.0%)	1.00
2	4(21.1%)	4(11.4%)	0.43
≥3	7(36.8%)	4(11.4%)	**0.04**
**Previous gastrectomy**			**0.01**
Yes	15(78.9%)	7(20.0%)	
No	4(21.1%)	28(80.0%)	
**PD-L1**			0.59
Positive	2(10.5%)	4(11.4%)	
Negative	3(20%)	4(11.4%)	
Unkown	14(69.5%)	27(77.1%)	
**Combined use**			
Combined with chemotherapy (XELOX/SOX/FOLFOX)		24(68.6%)	
Combined with target therapy		4(11.4%)	
Combined with chemotherapy and target therapy		5(14.3%)	
Combined with ipilimumab		2(5.7%)	

Data are number of patients (%) unless specified otherwise.

ECOG, Eastern Cooperative Oncology Group.

The meaning of the bold values was P value <0.05 and was considered statistically significant.

We also did subgroup analysis to compare the survival outcomes of monotherapy and combination therapy according to different stratifications (**Table 3**). In patients with liver metastases or elevated NLR levels (NLR above the median), anti-PD-1 administered in conjunction with other medications led to a more favorable mOS compared to those on monotherapy. (The HR in the subgroup with liver metastases was 0.33, 95% CI, 0.11–1.00, *P* = 0.05; HR in the subgroup with elevated NLR was 0.38, 95% CI, 0.14–1.00).

**Table 3:** Subgroup analysis of overall survival by monotherapy and combination therapy.

	Overall population	Monotherapy	Combination therapy
Tumor response data	(n=54)	(n=19)	(n=35)
Complete response	0	0	0
Partial response	9(16.7%)	2(10.5%)	7(20.0%)
Stabe disease	30(55.6%)	10(52.6%)	20(57.1%)
Progressive disease	15(27.8%)	7(36.8%)	8(22.9%)
Objective response rate, 95%CI	9(16.7%; 6.4-26.9)	2(10.5%; 1.9-29.6)	7(20.0%; 8.4-36.9)
Disease control	39(72.2%; 59.9-84.6)	12(63.2%; 38.4-83.7)	26(74.3%; 56.7-87.5)

Data are n (%) or n (%; 95% CI).

Of the five patients who accepted anti-PD-1 combined with apatinib, a small molecular anti-angiogenic agent, in the combination group, they all achieved a durable, stable disease, ranging from 4.20 to 9.37 months, although no objective response was observed.

### 3.3 Association between clinicopathological characteristics and survival outcomes

In univariate analyses with the Cox regression model, ECOG 0–1 was associated with better OS outcome (HR = 0.28, 95% CI, 0.11–0.56) (**Supplementary Table 1**). Patients with ascites and elevated NLR or PLR were at higher risk of death (HR for ascites was 3.27, 95% CI, 1.61–6.65, *P* = 0.001; HR for elevated NLR was 2.19, 95% CI, 1.18–4.05, *P* = 0.01; and HR for elevated PLR was 2.49, 95% CI, 1.33–4.65, *P* = 0.004). In light of the results from the univariate analyses, we selected the ECOG performance score, ascites, NLR, and PLR for multivariable analyses. ECOG performance score and ascites were independent risk factors associated with OS outcomes in patients treated with anti-PD-1-based therapy. NLR and PLR did not reach statistical significance in the multivariable analysis.

### 3.4 Safety and adverse events

TRAEs of any grade occurred in 27 patients (50%) in the overall cohort (**Table 4**). All-grade TRAEs observed in 5% or more of patients in the overall cohort included decreased neutrophil count, nausea, vomiting, increased alanine aminotransferase, and increased alkaline phosphatase levels. Patients treated with anti-PD-1 monotherapy (n = 5, 26.3%) had fewer TRAEs than those in the combination group (n = 22, 62.9%). There were also fewer patients with TRAEs of grades 3–5 with monotherapy (n = 2, 10.5%) than with combination therapy (n = 7, 20.0%). Grade 3 to 5 events were reported in nine patients (16.7%), including oral mucositis (n = 1, 5.3%) and gastric obstruction (n = 1, 5.3%) in the anti-PD-1 monotherapy group and decreased neutrophil count (n = 4, 11.4%), gastric perforation (n = 1, 2.9%), lung infection (n = 1, 2.9%), and pneumonitis (n = 1, 2.9%) in the combination group. Only one death was attributed to treatment in the combination group (one patient with gastric perforation). No deaths related to study treatment occurred in the monotherapy group. Pneumonitis was the only potentially immune-related adverse event reported in our cohort, which was observed in three patients (one with monotherapy and two with combination therapy).

**Table 4:** Safety and treatment-related adverse events.

		Monotherapy	Combination therapy	Hazard ratio(95%CI)	P value
	No. of patients	(n=19)	(n=35)		
Overall	54			0.70(0.36-1.35)	0.29
Gender					
Male	32	11	21	0.87(0.36-2.11)	0.75
Female	22	8	14	0.53(0.19-1.59)	0.24
Age					
<65	43	17	26	0.57(0.28-1.18)	0.13
≥65	11	2	9	1.40(0.16-12.05)	0.76
ECOG performance status					
0-1	36	8	28	0.80(0.31-2.11)	0.66
2-4	18	11	7	1.60(0.57-4.45)	0.37
Organs with metastases					
≤2	26	19	7	0.61(0.21-1.75)	0.36
>2	27	15	28	0.76(0.29-1.94)	0.56
Lymph node positive	47	18	29	0.79(0.39-1.60)	0.51
Peritoneum metastases					
Yes	31	13	18	1.12(0.42-2.30)	0.81
No	23	6	17	0.46(0.15-1.39)	0.17
Liver metastases					
Yes	27	5	22	**0.33(0.11-1.00)**	**0.05**
No	27	14	13	1.36(0.57-3.24)	0.48
Ascites					
Yes	25	10	15	1.00(0.40-2.00)	0.99
No	29	9	20	0.61(0.21-1.74)	0.35
Previous anticancer therapies for locally advanced/metastatic disease					
0-1	35	8	27	0.68(0.26-1.78)	0.43
≥2	19	11	8	0.60(0.20-1.79)	0.36
Previous gastrectomy					
Yes	22	15	7	0.59(0.16-2.12)	0.42
No	32	4	28	0.09(0.02-0.35)	0.00
Elevated LDH					
Yes	8	4	4	0.49(0.09-2.68)	0.41
No	46	15	31	0.76(0.36-1.62)	0.48
Elevated NLR					
Yes	26	9	17	0.38(0.14-1.00)	**0.05**
No	27	9	18	0.80(0.31-2.06)	0.64
Elevated PLR					
Yes	26	10	16	0.42(0.17-1.04)	0.06
No	27	8	19	0.84(0.29-2.44)	0.75
HER2 positive					
Yes	10	5	5	1.97(0.32-12.31)	0.74
No	27	11	16	0.72(0.29-1.79)	0.48

ECOG, Eastern Cooperative Oncology Group.

LDH, lactate dehydrogenase.

NLR, neutrophil to lymphocyte ratio.

PLR, Platelet to lymphocyte ratio. The meaning of the bold values was P value <0.05 and was considered statistically significant.

## 4 Discussion

To our knowledge, this was one of the first real-world studies that evaluated the performance of an anti-PD-1-based treatment regimen in the Chinese GC/GEJC population. Anti-PD-1 monotherapy and combination therapy showed favorable survival outcomes and manageable safety profiles in advanced or metastatic GC/GEJCs. The mOS was 11.10 months (95% CI, 7.05–15.15), 5.40 months (95% CI, 2.64–8.17), and 11.1 months (95% CI, 7.17–15.03) in the overall population, the monotherapy group, and the combination group, respectively, while the mPFS was 3.93 months (95% CI, 2.47–5.39), 2.93 months (95% CI, 0.85–5.01), and 4.07 months (95% CI, 1.83–6.03). Prolonged OS and PFS were observed in the combination group compared with the monotherapy group, although statistical significance was not reached. Objective response was achieved in 16.7%, 10.5%, and 20.0% of patients in the overall population, monotherapy group, and combination group, respectively. In the overall cohort, 72.2% of the patients achieved tumor regression or disease control (95% CI, 59.9–84.6%). In patients treated with anti-PD-1/L1 monotherapy as a third-line treatment, the mOS and mPFS were reported to be 4.6–5.6 months and 1.4–2.0 months, respectively (8–10). Among the patients treated as third-line or beyond treatment in our cohort, the mOS was 6.3 months (95% CI, 0–18.43) and the mPFS was 4.13 months (95% CI, 0.86–7.40). In phase II clinical trial, anti-PD-1 as a first-line treatment showed mOS of 13.8 months (95% CI, 8.6–not evaluable) in the monotherapy cohort and 20.7 months (95% CI, 9.2–20.7) in the combination cohort (21). Of the 14 patients in our cohort who received an anti-PD-1-based regimen as a first-line treatment, their mOS was 11.10 months (95% CI, not evaluable). Among these 14 patients, 4 (28.6%) had an ECOG performance score >1. That might explain the relatively inferior survival outcome compared with the previous report. In a phase Ib/II clinical trial, 18 Chinese metastatic GC patients received anti-PD-1 plus chemotherapy as first-line treatment, and 58 received anti-PD-1 monotherapy as second-line treatment. In the former cohort, mOS was not reached and mPFS was 5.8 months, while in the latter cohort, mOS was 4.8 months and mPFS was 1.9 months (22). In our cohort, 13 patients received anti-PD-1-based combination therapy as first-line treatment, with a mOS not reached and a mPFS of 5.2 months. Of the 18 patients who progressed after at least one systematic chemotherapy and were treated with anti-PD-1 monotherapy, the mOS was 6.3 months (95% CI, 0–15.54) and the mPFS was 2.87 months (95% CI, 0–5.91). Overall, our study showed results comparable to previously published interventional studies.

In a recent phase Ia/b clinical trial, 41 GC/GEJC patients whose disease progressed after one or two lines of systemic therapy were treated with pembrolizumab plus ramucirumab (an IgG1 VEGFR-2 monoclonal antibody). Of these patients, 21 (51%) achieved disease control, including 3 (7%) partial responses and 18 (44%), stable disease (23). In our cohort, five patients were given anti-PD-1 (three with pembrolizumab and two with nivolumab) combined with apatinib, and all five patients obtained clinical benefits with durable disease control. *De-novo* or acquired resistance to ICBs is complex and could be attributed to several factors, such as an immunosuppressive tumor microenvironment, a lack of PD-L1 expression, and T-cell exclusion (24–26). Anti-angiogenesis therapy could prevent immunotherapy resistance by increasing T-cell trafficking, migration across vascular endothelium, and infiltration into tumor tissue (27). Further studies with larger sample sizes are needed to verify the synergistic effects of anti-angiogenesis therapy with ICBs.

Patients recruited into randomized clinical trials are usually highly selected. Inclusion criteria typically include an ECOG performance score of <2 and no symptomatic brain metastases or other unfavorable physical conditions (28, 29). Randomized clinical trials provide an optimal way to evaluate the effectiveness and safety of a specific treatment. However, such studies are intrinsically less amenable to extrapolation. An ECOG performance score of >1 was previously reported as an independent risk factor for unfavorable survival upon treatment with ICBs in real-world studies (30, 31). In our cohort, of 18 patients with a baseline ECOG performance score of 2–4, only 5.6% (95% CI, 0–17.3%) obtained a clinical response. In contrast, for patients with a baseline ECOG of 0–1, the ORR was 22.2% (95% CI, 8.0–36.5%), and the mPFS was 5.20 months (95% CI, 2.03–8.37). Patients with ECOG 2–4 also had worse overall survival than those with ECOG 0–1 (median OS, 4.80 months vs. 13.23 months; HR = 3.54, 95% CI, 1.80–6.97). Consistent with previous studies, the ECOG performance score was validated as an independent OS risk factor (**Table 4**). Patients with brain metastases are usually excluded from randomized studies (9, 23). In our cohort, four patients had brain metastases at the onset of anti-PD-1 therapy; their mPFS was 1.30 months (95% CI, 1.05–1.56) and mOS was 8.37 months (95% CI, 0.98–15.75). In addition to the particular subpopulations mentioned above, there were nine patients in our study aged ≥70, among whom 10% responded to the treatment, and their mPFS and mOS were 3.07 months (95% CI, 2.66–3.48) and 5.17 months (95% CI, 4.58–5.75), respectively. In populations that usually do not meet the inclusion criteria of randomized studies, anti-PD-1-based therapy also showed favorable survival outcomes.

Various responses to ICBs by different tumor types mainly arise from the diversity of tumor immune microenvironments (32, 33). One of the well-investigated and commonly used biomarkers for systemic inflammatory response is circulating white blood cells, including neutrophils and lymphocytes (34, 35). Recent studies investigated the predictive value of NLR (neutrophil-to-lymphocyte ratio) and PLR for the treatment of checkpoint inhibitors. Low baseline NLR levels were associated with prolonged PFS and OS in metastatic melanomas following treatment with ipilimumab (CTLA-4) (36). In non-small cell lung cancers treated with anti-PD-1 inhibitors, low NLR and PLR levels were also reported to be associated with better PFS, OS, and response rate (37, 38). In our cohort, elevated NLR and PLR were predictive markers for OS in univariate analyses but not in multivariable analyses when considering clinical factors. This could have been partly explained by the small sample size, which might limit the statistical power.

In our cohort, combination therapy presented better survival outcomes in patients with liver metastases and elevated baseline NLR levels compared with anti-PD-1 monotherapy. The differences in PFS and OS between the treatment groups were no longer significant after adjusting according to clinical characteristics. In a recent update on the phase III study KEYNOTE-062, pembrolizumab combined with chemotherapy in gastric cancers as first-line therapy did not improve PFS or OS compared with pembrolizumab alone (15). In non-small cell lung cancer (NSCLC), anti-PD-1/L1 combined with chemotherapy showed better survival benefits than anti-PD-1/1 alone (39, 40). Tumor PD-L1 expression and the tumor immune microenvironment were previously reported to be affected by cytotoxic agents, which could explain the synergistic effects of the combination of anti-PD-1/L1 inhibitors and chemotherapy (41, 42). However, in the GC population, combination therapy’s synergistic effects were relatively modest. Significant spatial heterogeneity of genomic alterations and tumor immune microenvironment in GC might account for inconsistent results and should be considered in future clinical trial designs (43, 44). There are several limitations to our study. Firstly, the sample size was limited. However, to our knowledge, this is one of the first real-world studies to explore the effectiveness and safety of anti-PD-1/L1 inhibitor monotherapy and combination therapy as first-line or second-line treatment in GC, especially in Asian patients. Second, this is a retrospective cohort study and could have been potentially biased. However, a retrospective real-world cohort allows us to investigate whether patients with higher ECOG scores or more advanced GC could benefit from immunotherapies, which is usually not feasible with prospective trials. Moreover, patients were recruited consecutively in an effort to minimize bias, and comprehensive clinicopathological information was collected to enable adjustment in multivariable regression analyses. Third, the combination regimens were heterogeneous, and a specific combination regimen should be considered when designing prospective studies in the future. Furthermore, the real-world study was very different from prospective clinical trials, and the subjects included in the real-world study were highly heterogeneous. For patients with GC, the heterogeneity of the tumor itself and its impact on the patient’s physical condition, as well as their different physical status, treatment willingness, previous treatment history, economic status, drug availability, and other factors, affect the final treatment effect and survival outcomes. At the same time, due to the impact of the COVID-19 epidemic, the treatment of patients would be more or less affected, including whether they could come to the hospital to receive treatment during the prescribed time and the accessibility of drugs. The uncertainty of immunotherapy and the different methods of combination therapy also determined the heterogeneity and persistence of treatment.

On the other hand, real-world data played a vital role in perfecting and supplementing clinical research data. At the same time, we have seen whether immune monotherapy or combination therapy plays an important role, and the significance of this cannot be ignored in GC. Anti-PD-1 monotherapy and combination therapy showed promising survival outcomes and manageable safety profiles in advanced or metastatic GC/GEJCs in a real-world setting. Prolonged OS and PFS and increased ORR were observed in the combination group compared with the monotherapy group, although statistical significance was not reached. In some subgroups, such as patients with live metastases or elevated baseline NLR levels, combination therapy outperformed anti-PD-1 alone regarding survival outcomes. Treatment regimens involving anti-PD-1 in GC warrant further prospective investigations with larger sample sizes.

## Data availability statement

The original contributions presented in the study are included in the article/**Supplementary Material**. Further inquiries can be directed to the corresponding authors.

## Ethics statement

All participants in this study signed informed consent forms. The ethics committee of the People’s Liberation Army General Hospital approved this study according to the ethical standards of the Declaration of Helsinki and its subsequent amendments(Ethical approval number:S2020-284-01). The patients/participants provided their written informed consent to participate in this study. Written informed consent was obtained from the individual(s) for the publication of any potentially identifiable images or data included in this article.

## Author contributions

TaL, TiL, and LZ served as co-first authors, with primary contributions to the manuscript. LL, XZ, and JW contributed to the acquisition, analysis, and interpretation of data. Study supervision: YH, FZ. All authors contributed to the article and approved the submitted version.

## Supplementary material

Supplementary Figure 1 Kaplan-Meier plot of overall survival in patients treated as first and second-line therapy.

Supplementary Figure 2 Kaplan-Meier plot of overall survival in patients treated as third-line or beyond therapy.

Supplementary Figure 3 Kaplan-Meier plot of progression-free survival in patients treated as first and second-line therapy.

Supplementary Figure 4 Kaplan-Meier plot of progression-free survival in patients treated as third-line or beyond therapy.

Supplementary Table 1 Univariate analysis and multivariate analysis for overall survival
