# Supplementary figures and images for: Effectiveness and safety of anti-PD-1 monotherapy or combination therapy in Chinese advanced gastric cancer: A real-world study
Source: Front Oncol. 2023 Jan 5;12:976078. doi: 10.3389/fonc.2022.976078 (PMC9850086; doi:10.3389/fonc.2022.976078)

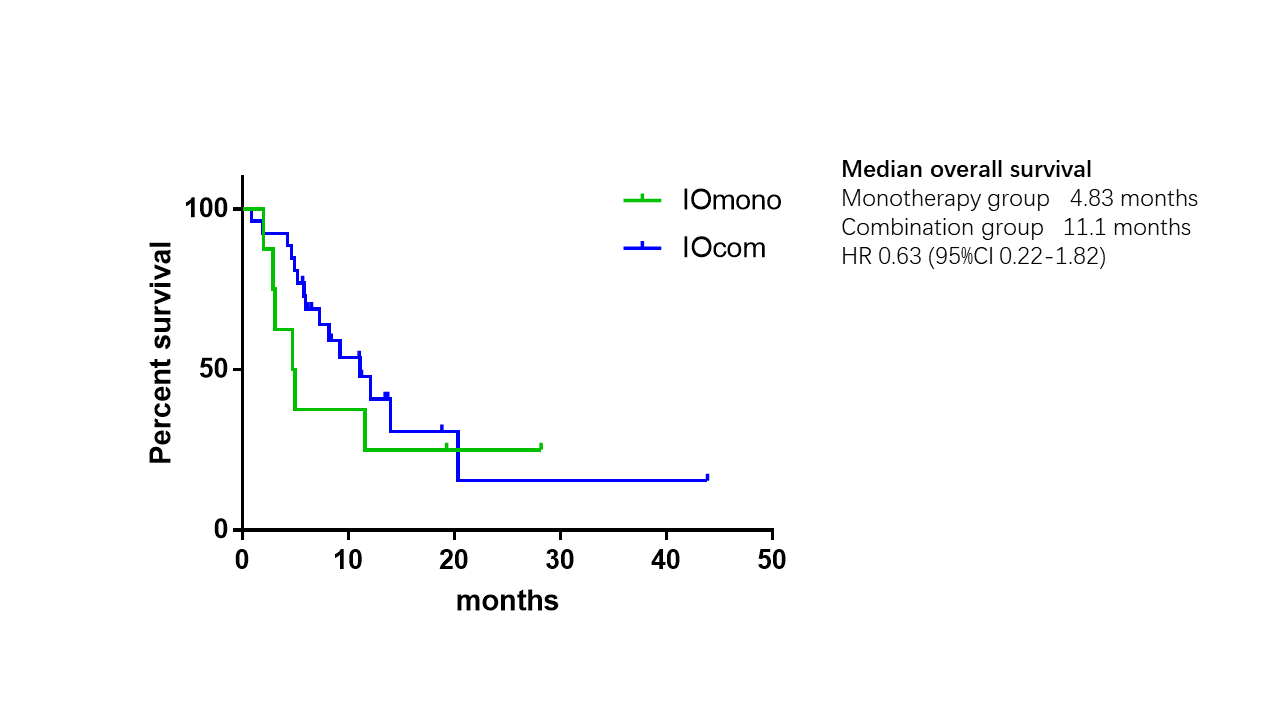

Supplement: Supplementary Figure 1 — Kaplan-Meier plot of overall survival in patients treated as first and second-line therapy. [file Image_1.tif]

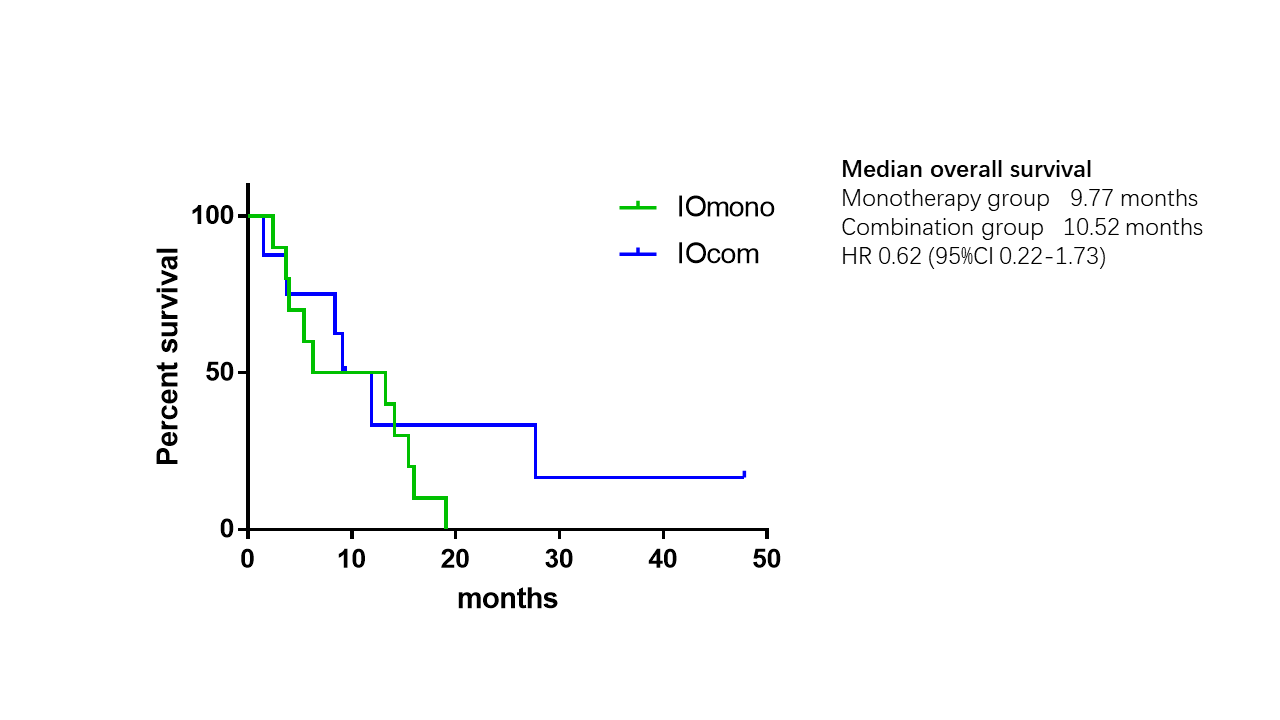

Supplement: Supplementary Figure 2 — Kaplan-Meier plot of overall survival in patients treated as third-line or beyond therapy. [file Image_2.tif]

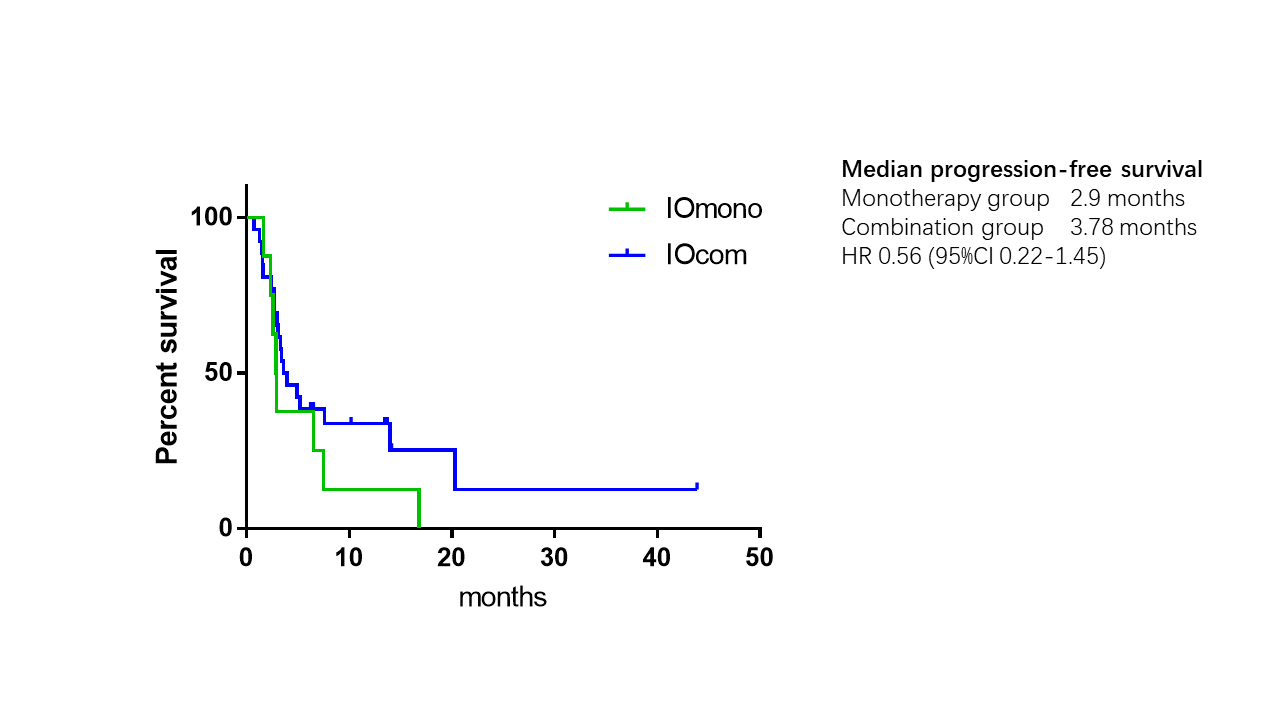

Supplement: Supplementary Figure 3 — Kaplan-Meier plot of progression-free survival in patients treated as first and second-line therapy. [file Image_3.tif]

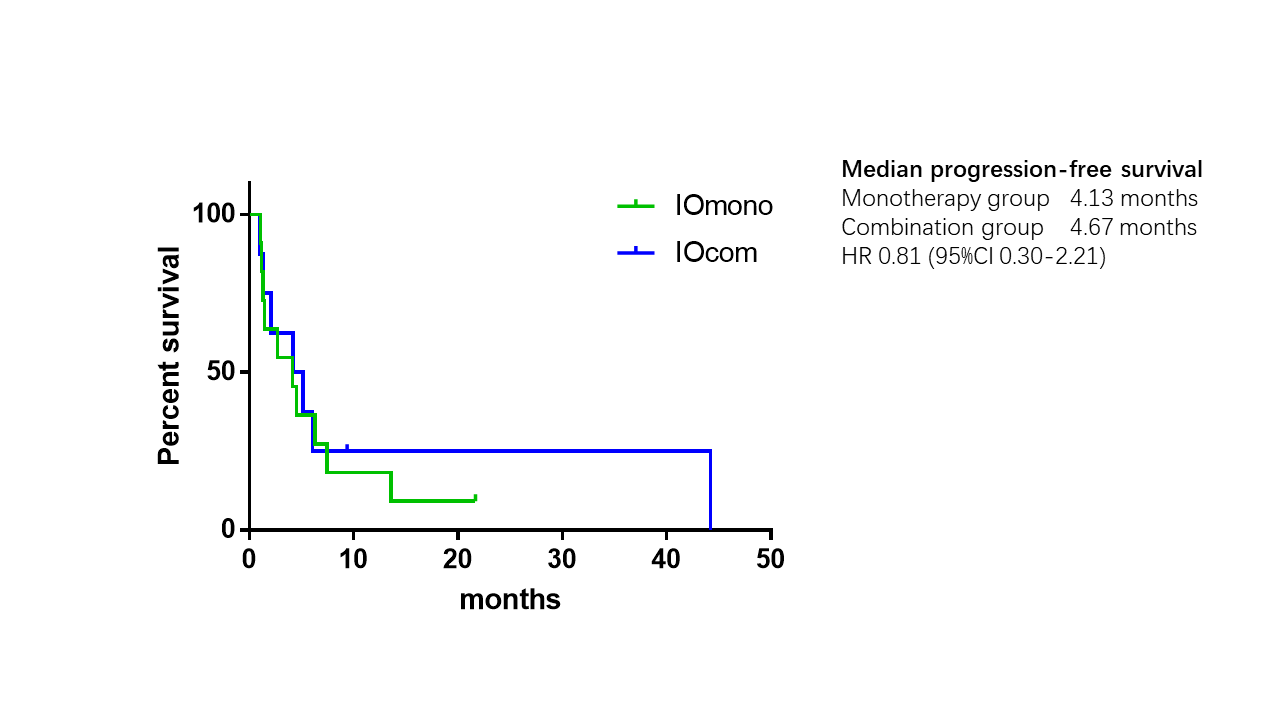

Supplement: Supplementary Figure 4 — Kaplan-Meier plot of progression-free survival in patients treated as third-line or beyond therapy. [file Image_4.tif]
